# Experiences and Impact of Stigma and Discrimination among People on Antiretroviral Therapy in Dar es Salaam: A Qualitative Perspective

**DOI:** 10.1155/2016/7925052

**Published:** 2016-03-24

**Authors:** Maisara Mhode, Tumaini Nyamhanga

**Affiliations:** ^1^School of Nurse Teachers, Muhimbili University of Health and Allied Science, Dar es Salaam, Tanzania; ^2^Department of Development Studies, Muhimbili University of Health and Allied Sciences, Dar es Salaam, Tanzania

## Abstract

*Background*. The impact of stigma on adherence to antiretroviral therapy (ART) has been less studied in Tanzania. Recent studies indicate that people on ART still experience stigma. Qualitative information on the subject matter is especially insufficient.* Objective*. This paper reports on the dimensions of stigma and discrimination and their impact on adherence to ART as experienced by people living with HIV (PLHIV).* Design*. A phenomenological approach was used to gather information on the lived experiences of stigma and discrimination. The sample size was determined according to the saturation principle.* Results*. Respondents experienced different forms of HIV-related stigma such as verbal, social, and perceived stigma. Various forms of discrimination were experienced, including relational discrimination, mistreatment by health care workers, blame and rejection by spouses, and workplace discrimination. HIV-related stigma and discrimination compromised ART adherence by reinforcing concealment of HIV status and undermining social suppport.* Conclusion*. After nearly a decade of increasing the provision of ART in Tanzania, PLHIV still experience stigma and discrimination; these experiences still appear to have a negative impact on treatment adherence. Efforts to reduce stigma and discrimination remain relevant in the ART period and should be given more impetus in order to maximize positive treatment outcomes.

## 1. Introduction

HIV/AIDS is a major public health concern in sub-Saharan Africa where 69% of all PLHIV are found [1, 2]. Furthermore, the region accounts for three-quarters of AIDS deaths. In response to this ugly situation, a number of countries in the region, including Tanzania, have embarked on the provision of antiretroviral therapy [3].

Tanzania started to increase the provision of antiretroviral therapy in 2004 [4]. It is estimated that between 150,000 and 200,000 people are currently receiving ART, which represents between 63% and 83% of those needing it [4]. The long term success of this care and treatment programme depends on understanding the factors influencing adherence to the treatment [5]. The global literature shows that optimal adherence to treatment has been found to be influenced by a number of factors which are summarised into four main groups: (1) patient factors, (2) medication factors, (3) the patient-health care provider relationship, and (4) the system of care [5–8].

The impact of stigma on adherence has been less studied, particularly in Tanzania. Recent studies indicate that people on ART still experience stigma [9–16]. Qualitative information on the subject matter is especially insufficient. This paper, therefore, reports on the dimensions of stigma and discrimination and their impact on adherence to antiretroviral therapy (ART) as experienced by people living with HIV (PLHIV).

*Conceptual Framework*. In this paper, HIV/AIDS-related stigma is defined as an attribute or quality which “significantly discredits” PLHIV who are on ART in the eyes of their family, community, and health care providers [17]. HIV/AIDS-related discrimination implies denying PLHIV the necessary social support for the consistent uptake of antiretroviral drugs.

Deacon [18] argues that there are different types of stigma associated with HIV/AIDS, namely, self-stigma, anticipated stigma, and enacted stigma. Self-stigma occurs when an individual internalizes feelings of shame or blame due to his/her negative social judgment of the HIV positive status. Anticipated stigma is a negative response PLHIV expect to receive from their family and community if their HIV positive status was made known. Enacted stigma is discrimination which involves actual acts or omissions that disadvantage a person on ART.

There are several social forces that determine stigma, including gender, poverty, and other social inequalities [19]. For instance, gender inequality determines the extent to which sexism will mark the course of the HIV disease; for example, in a highly patriarchal society, the disclosure of HIV positive status is more likely to provoke stigma and the threat of domestic violence, than in settings where women enjoy gender equity. Poverty, which by itself is an undesirable attribute, could be the primary cause of greater stigma among PLHIV. Unfortunately, gender inequality and poverty exacerbate one another, thereby synergistically increasing the risk of AIDS-related stigma. It follows that fear of stigma and discrimination results in nondisclosure of an HIV positive status which, in turn, affects adherence to treatment. Likewise, HIV-related stigma and discrimination diminish social support for PLHIV, thereby increasing the risk of poor adherence to antiretroviral therapy.

## 2. Method

### 2.1. Study Site

The study took place at the Mnazi Mmoja Care and Treatment Clinic (CTC) which is situated in Ilala Municipality, Dar es Salaam. The Dar es Salaam region was chosen because it was the first to implement the provision of free ART in June, 2004, with a pilot programme at Muhimbili National Hospital, after which the programme of care and treatment started in Tanzania [4]. Since then, care and treatment have expanded to cover the entire country.

### 2.2. Study Design

This was a qualitative study which used a phenomenological approach to gather information on the lived experiences of stigma and discrimination. The phenomenological approach seeks to describe the meanings embedded in the human experience so as to understand the essence of the phenomena being investigated [20].

### 2.3. Study Population

The population studied included everyone living with HIV and AIDS, 18 years and above, attending the CTC clinic at Mnazi Mmoja, and on ART.

### 2.4. Sample Size

The sample size was determined according to the saturation principle; that is, data collection stopped at the point when new data no longer brought additional insights into the research questions [21]. A total of 26 PLHIV who were on ART participated in this study. Characteristics of respondents are shown in Table 1.

### 2.5. Sampling Procedure

A nonprobability purposive sampling method was used to select the study respondents. HIV positive people who had been on ART for more than two months were purposively selected, because they had experience of the situation and were able to give more information concerning stigma and its impact on adherence to antiretroviral therapy. The clinic in charge was briefed about the study and asked to identify potential respondents.

### 2.6. Data Collection Procedures

Data were collected by means of in-depth interviews (IDIs). These were unstructured and guided by open ended questions. Through this guide, an attempt was made to explore respondents' experiences on various dimensions of stigma and how they impacted on treatment adherence. It is worth noting that the questioning did not necessarily follow a sequential order [22]. That is, the order in which the questions were asked varied in response to interaction with the informants. The language for the interviews was Kiswahili. The interviews were audio-recorded, transcribed, and then translated into English. The IDIs took place in a private and quiet place to maximise privacy and lasted for 30 to 45 minutes.

### 2.7. Ethical Approval

Ethical approval was sought from the Institutional Review Board of Muhimbili University of Health and Allied Sciences. After the study received ethical clearance, permission to conduct the study was also sought and received from the Ilala Municipal Authority. Each potential respondent was informed about the main objective of the study and about his or her right to decline participation outright, or to withdraw consent at any stage of the research, without undesirable consequences. If the potential respondent agreed to participate in the study, he or she was asked to sign a consent form. Data were gathered without the researcher knowing the names of respondents or being made aware of any unique identifiers attached to the data.

### 2.8. Data Analysis

A thematic analysis approach was used to analyze the data; the use of this approach implies that data were analyzed through the examination and categorization of respondents' comments. The analysis was carried out in three stages: firstly, by line-by-line coding of field notes and transcripts; secondly, by in-depth examination and interpretation of the resultant codes and their categorization into descriptive and analytical themes; and, thirdly, by development of an overarching theme [23, 24]. The coding involved the development of concepts; that is, the data were pierced into discrete elements in order to expose underlying thoughts and meanings.

## 3. Results

### 3.1. Sociodemographic Characteristics of the Respondents

As summarised in Table 1, most respondents (16 out of 26) were females and belonged to the age group of 31–40 years. About half (12 out 26) of the respondents had a primary level of education or had never gone to school. Most (14 out 26) had a low level of income, of whom 11 engaged in small scale business and three were not employed at all.

### 3.2. Themes

The themes for this study emerged out of the thematic network analysis process (see Table 2). The process generated 23 codes covering nine basic themes. These basic themes were further clustered into eight organising themes, which were finally condensed into four main (global) themes. These major themes are

(i) dimensions of the stigma experience among people on ART;
(ii) dimensions of the discrimination experience among people on ART;
(iii) HIV-related stigma and discrimination compelling PLHIV not to adhere to antiretroviral therapy;
(iv) coping strategies adopted to deal with HIV/AIDS stigma and discrimination.

These themes are relevant to the focus of the study and serve as key subheadings in the sections that follow.

### 3.3. Dimensions of the Stigma Experience among People on ART

Different forms of HIV-related stigma among people on ART were experienced by respondents, including verbal stigma, social stigma, and perceived stigma (the fear of stigma associated with disclosing the HIV status).

#### 3.3.1. Verbal Stigma

Participants reported that if one is suspected of being HIV positive and on ART, he/she is given names that imply that his/her days of staying alive are numbered. The stigmatising names included phrases such as “keys to mortuary.” This is what one of the respondents said:

*Sometimes it is not the disease that kills…it is the bad words and remarks from people. One day I heard one person telling a patient who was on ART that she was key to mortuary because he believed that, once you are on ART you are about to die. (Female respondent, 34 years old)*

This tendency of labelling people living with HIV, or name calling, is part of gossip and accounts for most of the stigmatising behaviours from the community. The participants argued that labelling is conceptualised as society's symbolic punishment for those who are considered to have violated sexual moralities.

#### 3.3.2. Social Stigma

The respondents expressed fear of being morally judged for being HIV positive and on ART. Their fear was based on the observation that community members tend to associate HIV positive status with engagement in immoral behaviours, as expressed by one of the respondents:

*I was scared because I was worried about the response of my partner and other people close to me. I was concerned that they would want to know how I got infected. So I asked myself, how will my husband regard me? Will he understand me? But I believe I got infected with HIV when I had a blood transfusion. I have never been a prostitute. (Female respondent, 35 years old)*

A similar concern was expressed by another respondent:

*It really pained me and I cried a lot. I asked myself, where did I get this disease? But then I realised that I got it from my parents. One day I went to visit my aunt and I saw my young brother (who was taken in by my aunt after the death of our mother) using antiretroviral drugs. I asked my aunt and then I knew I also got HIV from our parents. I noticed I'm HIV positive in 2006 but I did not let anybody know because I feared how they would regard the circumstances that made me get infected. (Female respondent, 23 years old)*

The participants, particularly women, attributed social stigma to community members' perceptions that all HIV positive people were prostitutes or engaged in sex carelessly and that being on medication means one is about to die.

#### 3.3.3. Perceived Stigma

Respondents expressed a fear of being stigmatised; that is, they felt that they might be treated negatively by family and community members, as expressed by one of the respondents:

*I have not taken my medication on two occasions when relatives and neighbours were in my house and have never disclosed my status to them. I did not have time to take out the medicine from my drawer. I frequently face this problem of trying to hide my medicine from others because I am living in a rented single room and I feared if they see my ARVs they will tell others I am infected. (Male respondent, 42 years old)*

Respondents feared negative consequences if their HIV status became known to their close relatives. They had experienced friends and relatives gossiping and ostracising people living with HIV. Indeed, it was reported that the fear of stigma prevented some users of antiretroviral drugs (ARVs) from seeking support, even when they were seriously sick, and this also may affect their adherence to ARVs:

*The society does not know my HIV status because I have not told them and will not, since they have a negative attitude towards people on ARVs. One day, when I came to clinic, there was a woman who was very sick who stays in Temeke but she had come alone [to a clinic in a neighbouring district of Ilala] because she did not want anyone to know where she was going. People are still isolating us. (Female respondent, 42 years old)*

### 3.4. Dimensions of Discrimination Experience among People on ART

The respondents experienced various forms of discrimination, including relational discrimination, mistreatment by health care workers, blame and rejection by spouses, and workplace discrimination.

#### 3.4.1. Relational Discrimination

Respondents experienced changes in relationships when spouses, family members, friends, and neighbours discovered that a particular person was on ART. This was well narrated by one of the respondents:

*One day when I came back from work I saw something unusual from my neighbours. I thought about the situation for sometime but didn't get an answer. Later, one of the neighbours came and told me what happened a few hours ago. She told me that my son came out with my ARV container thinking that it was children's play device [locally-manyanga] because when the ARVs are in a tin and shaken, they produce a certain sound. That day, I forgot the keys of the drawer where I usually keep my medicines. One of my neighbours told my son to give the medicines to her and she went to the pharmacy to confirm if they were truly ARVs or not. After confirming, she brought them back to my son and from that day our friendship changed, because previously we used to cook and eat together, but nowdays [sic] she is no longer interested in sharing anything with me…. She told other neighbours about it and caused me to be very unhappy. (Female respondent, 36 years old)*

#### 3.4.2. Spousal Discrimination

Respondents complained of blame and less support from their partners once diagnosed with HIV and because of the fact that they were on ART. In spite of a reduction of internalised stigma, some respondents experienced blame-related stigma from their spouses and their communities. This was well narrated by one of the respondents:

*I was told I have HIV and I had to start treatment. I told my husband, but suddenly he left and came back after a week and divorced me. He told me to go to my relatives so that they could take care of me when I fall sick because he blamed me as the source of the problem [she spoke with grief]. (A female respondent, 39 years old)*

A similar concern was expressed by a male respondent:

*My wife is the one who brought the disease. I warned her many times about infidelity but she did not care. When I told her that I'm HIV positive she deserted me and went to her parent's home. She left me with a 3 year old child. (Male respondent, 49 years old)*

When stigma was experienced within the family, its consequences could be harder to overcome and even resulted in treatment interruption.

#### 3.4.3. Mistreatment by Health Care Workers

Respondents reported that sometimes they experienced discrimination from health care providers, manifested by neglect, verbal abuse, overusing protective materials such as gloves, and paying little attention to their concerns. The following quote is illustrative:

*Sometimes, if you ask health care providers something, they do not respond to your question. They pretend to be busy, though most of the time it is not true that they are busy, they just neglect us. Sometimes they use abusive language against us and they delay giving services. They don't value people on ART anymore in this world. When you arrive late to the clinic you are told that you will be moved closer to where you live. They use it as a way to threaten us because they know most of us are going away to avoid discrimination. (Male respondent, 43 years old)*

Four respondents complained about the care they were given at Mwananyamala CTC (the clinic they used to attend before moving to Mnazi Mmoja CTC). Nurses at Mwananyamala CTC were accused of doing inhuman things that influenced respondents' decisions to move to Mnazi Mmoja. For instance, one of the respondents claimed the following:

*At Mwananyamala CTC some nurses delay in attending to patients and use offensive statements. One day, a nurse told me “take your ‘bomb'” - meaning my file - and she continued, saying ‘I am not the one who gave you HIV'. Whenever I see that nurse or remember those words - it hurts a lot [the participant cried after narrating that painful experience]. (Female respondent, 49 years old)*

### 3.5. Impacts of Stigma and Discrimination on Adherence to Antiretroviral Therapy

The fear of stigma experienced by people on ART resulted in nonadherence to medication through a number of ways. Firstly, because of stigma, a substantial number of respondents revealed that they attend a care and treatment clinic that is far away from their homes. They fear being identified by people who know them, including health staff at a nearby CTC, as pointed out by one of the participants:

*No. I do not like the nearby clinic, because many people know me well and can see me. And even healthcare provider sat the hospital near to where I live have a tendency to break patient confidentiality. A good example is one of my friends who was declared publicly at a barto be HIV positive and she is now on medication [ARVs]. (Female respondent, 37 years old)*

Consequently, a patient may feel compelled to go to a different district, or even region. For instance, a person living in Mbagala in the Temeke District would prefer to go to Mnazimmoja CTC, which is in a neighbouring district of Ilala. Indeed, one of the respondents in this study lived in the Morogoro region. She decided to come all the way (193 Km) to Dar es Salaam in an attempt to conceal her HIV status from her neighbours:

*I live in Morogoro but I usually refill my ARVs here at Mnazi Mmoja CTC. So, sometimes I run short of ARVs because of inability to come to refill in Dar es Salaam. (Female respondent, 23 years old)*

A similar concern was expressed by another respondent:

*I sometimes worried about meeting my neighbours in hospital for ARV refills. What I am doing nowdays [sic] is to attend a far away clinic and wear a hijjab and sun-glasses which will not be easy for whoever knows me to recognise who I am. I am not a muslim and am not wearing this in my community. I always carry them in my bag and once I see an ATM machine I wear them there, then I go to the clinic, and I do the same once I want to go back home/to work. All the time I worry. One day I did not refill my ARVs due to bumping into relatives. (Female respondent, 35 years old)*

Consequently, because of preferring a distant CTC, some ART clients may fail to attend regularly, partly due to lack of the fare for transport, and thereby run short of ARVs.

Secondly, stigma reinforces the concealment of HIV status and consequently one may not feel at ease to take ARVs in the presence of family members or work colleagues, thereby delaying ingesting the drugs. The following remark made by one of the respondents is illustrative:

*I have not taken my medication for two occasions when relatives and neighbours were in my house and I have never disclosed my status to them. I did not get time to take out medicine from my drawer. I frequently face this problem of trying to hide my medicine from others because I am living in a rented single room and I feared if they see my ARVs they will tell others I am infected. (Male respondent, 42 years old)*

Another participant added the following:

*I am not free because I have not told people that I am sick. I have a single room. As a result, sometimes I delay taking my dose of ARVs as I have to wait for people to leave so that I remain alone. Similarly, at work I tend to go the toilet to take medications because I do not want them to know or suspect anything about my HIV status. (Male respondent, 24 years old)*

Thirdly, HIV-related stigma and discrimination undermine social support which often culminates in food insecurity and attendant fears to take ARVs on an empty stomach. One of the respondents remarked the following:

*ART helps me. But what makes me deteriorate nowadays is the inconsistent availability of food. I don't get enough food and fruits as I was advised. I am not capable of buying food. I am worried about the side effects of taking medicine on an empty stomach. (Male respondent, 49 years old)*

### 3.6. Coping Strategies Adopted to Deal with HIV/AIDS Stigma and Discrimination

Key coping mechanisms that emerged from respondents' narrations included spiritual devotion, acceptance of the illness, seeking information and/or exchanging views about the illness, preemptive disclosure, putting ART in an unlabelled envelope, and swallowing ARVs in the wash room [toilet].

#### 3.6.1. Spiritual Devotion

The majority of respondents indicated that their faith in God gave them courage to adhere to medication. They received spiritual and psychological support from the religious leaders. Spiritual devotion appeared to be a strong coping strategy among study respondents:

*I pray to God everytime [sic] I take my medicine and I believe one day Jesus Christ will cure me. (Female respondent, 30 years old)*

The study revealed that respondents' belief in the healing power of God had better health outcomes as their belief reduced their self-stigma. Indeed, their level of faith in the healing power of God was so high that some thought that, even if they died, it would not be because of HIV; it would be God's will. The following quote is illustrative:

*I get comfort from my religion because God is the one who enables us to live. In general, I perceive AIDS as a common disease and death is not necessarily caused by AIDS. You can sleep without being sick and still die. So I believe if I die, then it is God's will and not AIDS. (Male respondent, 41 years old)*

#### 3.6.2. Acceptance of the Illness

The study respondents accepted the condition of being HIV positive and considered HIV/AIDS as no longer fatal, particularly after they started ART and experienced a remarkable improvement in health. One of the respondents shared his feelings as follows:

*I see HIV as any other disease and I do not think that I will die because of it. When I die, I will know it is God's will, not because of AIDS. If I were to die, I could have died when I was very sick those days. (Male respondent, 57 years old)*

A similar concern was expressed by another respondent:

*My conscience has agreed to the problem so I regard it as just any other common disease. (Female respondent, 30 years old)*

The notion of regarding HIV/AIDS as an ordinary disease has been internalised mainly by the respondents who were on ART for three years or more. Some respondents spoke of the support they received from their families as having contributed to the peace of mind they were experiencing.

#### 3.6.3. Concealing the Identity of ARVs

Respondents stated that, in the face of stigma and discrimination, they are compelled to conceal the identity of ARVs by putting them in an unlabelled envelope, or one bearing a drug name that is unfamiliar to most lay people. This measure was well expressed by one of the respondents:

*I remove ARVs from their original container and put them in a plain envelope from where I take them, even in the presence of other people. (Female respondent, 28 years old)*

#### 3.6.4. Sharing Experiences about Taking ARVs in Support Groups

Social support groups seemed to play an important role in handling stigma and discrimination. Such groups constitute a forum for learning about challenges related to living with HIV and taking ARVs.

The following quote is illustrative:

*We discuss with my fellow support group members different challenges related to taking ARVs and how to cope. In this way, we comfort each other. We meet weekly in our support group. Also, my wife comforts me. She is a hospital employee so she knows these things well. (Male respondent, 52 years old)*

#### 3.6.5. Preemptive Disclosure

Some people on ART disclose their HIV status to family members, neighbours, and/or work colleagues and talk very freely about it and the challenges they face, so preempting gossip. This coping mechanism was well narrated by one of the respondents:

*I have disclosed my status to almost everybody because I am sometimes seen on the television or heard on the radio revealing that I am HIV positive (I am an AIDS activist). At my workplace (school) everybody knows I am infected. It helped me a lot to disclose my status because I sometimes feel unable to work and I just tell my boss by phone that I am sick. He always understand the situation and my colleagues say jokingly “mmmh! today viruses have woken up and are harassing her (in local language - virusi vimekolokochoa”) that's why she was not able to report to work. One day, one of my colleagues told me that some of teachers were saying that I didn't come yesterday because viruses were harassing me, but I just told her, I don't care (she laughs). (Female respondent, 46 years old)*

People of this kind, who disclose and talk freely about their HIV+ status, can strengthen others living with HIV by helping them overcome internalised stigma, cope with stigma, rebuild their self-esteem, and develop skills to take leadership roles in antistigma education and action.

## 4. Discussion

A range of ways in which stigma and discrimination affect ART adherence were found in this study. Many were consistent with those found in other studies, and others were new and unique to this setting. This section is organised into three subsections. The first section discusses dimensions of stigma and discrimination among people on ART. The second concerns the impact of stigma and discrimination on ART adherence. The third discusses coping mechanisms.

### 4.1. Dimensions of Stigma and Discrimination among People on ART

The findings of this study indicate that the respondents experienced different forms of HIV-related stigma, including verbal, social, and perceived stigma (the fear of stigma associated with disclosing HIV status). This suggests that both intrapersonal and interpersonal fears still exist, despite nearly a decade of increasing the provision of ARV. The study respondents experienced loss of dignity and friendship. Similar findings have been reported in other countries [25]. The respondents experienced various forms of discrimination, including relational discrimination, blame and rejection by their spouses, workplace discrimination, and mistreatment by health care workers. This suggests that, despite improvements in health status as a result of being on ART, PLHIV are denied services or entitlements as a result of deliberate actions or omissions by spouses, family members, friends, and/or health care workers. These findings are supported by several other studies [16, 26–30].

### 4.2. The Impact on ART Adherence of Stigma and Discrimination

The study found that the fear of stigma experienced by people on ART results in nonadherence to medication through a number of ways. Firstly, it was noted that patients prefer a distant CTC to the extent of avoiding CTC(s) available near to their homes, thereby risking irregular replenishment of their ARVs. This is because they fear being seen by people who know them as friends and neighbours. Similar findings have been reported in other African countries, where many respondents were unwilling to seek treatment at the nearest health facility [31–33]. Secondly, the study has shown that stigma reinforced the concealment of HIV status. Not wanting to tell others about an HIV positive status has been found to be a major impediment to the optimal uptake of ARVs. These results are in agreement with those reported by other scholars [34–36]. Likewise, Nyamhanga [37] found that nondisclosure of HIV+ status to the spouse was due to fear of violence and divorce/separation and it affected some women's attendance of CTCs, as they lacked the fare and were unable to justify their absence from home on the clinic day. Thirdly, this study found that stigma and discrimination undermined social support which, in turn, makes the person on ART vulnerable to food insecurity. The relationship between food insecurity and suboptimal adherence has been reported in Kenya [38] where respondents had a fear of taking ARVs on an empty stomach, because they were considered to be highly toxic drugs.

#### 4.2.1. Coping Strategies Adopted to Deal with HIV/AIDS Stigma and Discrimination

Key coping mechanisms that emerged from the respondents' narrations included becoming secretive, spiritual devotion, acceptance of the illness, seeking information and/or exchanging views about the illness through support groups, and preemptive disclosure. These coping mechanisms can be placed into two categories, namely, adaptive and maladaptive strategies.

Spiritual devotion, acceptance of the illness, seeking information and/or exchanging views about the illness, and preemptive disclosure can be considered as adaptive mechanisms because they foster positive living. These findings are consistent with those reported by Makoae et al. [39] who examined how PLHIV cope with HIV-related stigma in the five Southern African countries of Lesotho, Malawi, South Africa, Swaziland, and Tanzania. However, choosing to be secretive is maladaptive [unproductive], as it may result in an impediment to effective access and adherence to ART, as reported by Lekganyane and du Plessis [25].

### 4.3. Study Limitation

Since the study was qualitative in nature and therefore involved the purposive selection of the study sample, its findings cannot be generalised. However, being a qualitative study, its goal was not to generalise but rather to provide rich information on the dimensions of stigma and discrimination experienced by PLHIV on ART.

### 4.4. Conclusion and Recommendations

After nearly a decade of increasing the provision of ART in Tanzania, PLHIV still experience verbal and social stigma, relational discrimination, blame and rejection by spouses, and/or mistreatment by health care workers, albeit to a smaller extent compared to the pre-ART era. These unhealthy experiences have a negative impact on treatment adherence. Efforts to reduce stigma and discrimination that started earlier (in the pre-ART period) with the purpose of encouraging HIV testing are still relevant now and should be given more impetus so as to maximise positive treatment outcomes. Such efforts should contribute to helping ART clients apply adaptive coping strategies, such as those identified in this study that include accepting the illness, seeking information and/or exchanging views about the illness through support groups, and preemptive disclosure.

## Figures and Tables

**Table 1:** Summary of sociodemographic characteristics of study participants.

Characteristics	Number of participants
Age (years)	
21–30	**4**
31–40	**12**
41–50	**6**
51–60	**3**
61–70	**1**
Total	26
Sex	
Male	**10**
Female	**16**
Total	26
Level of education	
None	**2**
Primary	**10**
Secondary O-level	**11**
Secondary A-level	**1**
Degree	**2**
Total	26
Marital status	
Single	**6**
Married	**17**
Divorced	**2**
Separated	**1**
Total	26
Occupation	
Unemployed	**3**
Self-employed	**2**
Housewife	**3**
Government-employed	**1**
Small scale business	**11**
Others	**6**
Total	26

**Table 2:** Coding of text data and development of themes.

Text	Codes	Basic themes	Organising themes	Global themes

*Sometimes it is not the disease that kills…it is the bad words and remarks from people. One day I heard one person telling a patient who was on ART that she was “key to mortuary” because he believed that, once you are on ART you are about to die.*	(i) Gossip (ii) Bad words	(i) Name calling against PLHIV	Verbal stigma	Forms of stigma against people on ART
*I was scared because I was worried about the response of my partner and other people close to me. I was concerned that they would want to know how I got infected. So I asked myself how will my husband regard me? Will he understand me? how will he judge me? But I believe I got infected with HIV when I had a blood transfusion. I have never been a prostitute.*	(i) Worry (ii) Fear of how they contacted HIV	Concern about perception by significant others	Social stigma	
*I did not take my medication on two occasions when relatives and neighbours were in my house and have never disclosed my status to them. I did not get time to take out medicine from my drawer. I frequently face this problem of trying to hide my medicine from others because I am living in a rented single room and I feared if they see my ARVs they will tell others I am infected.*	(i) Judgement (ii) Feeling too insecure to take medicine openly (iii) Fear of disclosing status (iv) Fear of being seen taking ART (v) Hiding medicine (vi) Fear of telling others about infection	Afraid of being stigmatised	Perceived stigma	

*One of my neighbours told my son to give the medicines to her and she went to the pharmacy to confirm if they were truly ARVs or not. After confirming, she brought them back to my son and from that day our friendship changed, because at first we used to cook and eat together but nowdays [sic] she is no longer interested in sharing anything with me.*	(i) Change in relationship (ii) Isolation (including physical and social exclusion)	(i) Fear of being morally judged for being HIV positive and the fact that one is on ART	Relational discrimination	Dimensions of discrimination experience among people on ART
*I was told I have HIV and I had to start treatment. I told my husband, but suddenly he left and came back after a week and divorced me. He told me to go to my relatives so that they can take care of me when I fall sick because he blamed me as the source of the problem.*	(i) Blame (ii) Harassment (iii) Desertion	Blame and rejection of the spouse	Spousal discrimination	

*I have not taken my medication at the right time on two occasions because relatives and neighbours were in my house and I didn't disclose my status to them.*	(i) Not taking pills at the right time (ii) Concealment	(i) Open taking of ARVs equated to disclosure of HIV+ status which is unwanted (ii) Concealment of HIV status robs PLHIV of freedom to take ARVs in the presence of family members or workmates, thereby delaying ingesting the drugs	(i) Stigma reinforces concealment of HIV status (ii) Lack of freedom to take ARVs	HIV-related stigma and discrimination compel PLHIV not to adhere to antiretroviral therapy
*I am living in Morogoro but I usually refill my ART here at Mnazi Mmoja CTC. So, sometimes my ART might be finished but I don't have the bus fare to come to refill in Dar es Salaam.*	(i) Lack of bus fare (ii) Failure to refill ARVs	Avoidance of CTCs within the district of residence for fear of raising suspicion over HIV+ status to relatives, neighbours, and friends	Failure to disclose for fear of being discriminated against	

*I pray to God everytime [sic] I take my medicine and I believe one day Jesus Christ will cure me.*	(i) Religious belief	Role of spiritual healing	Spiritual devotion	Coping strategies adopted to deal with HIV/AIDS stigma and discrimination
*My concience [sic] has agreed to the problem so I regard it asjust [sic] any other common disease.*	(i) Spiritual healing			
*We discuss with my fellow patients different HIV issues and give comfort to each other. We meet weekly in our support group. Also, my wife comforts me. She is a hospital employee so she knows these things well.*	(i) HIV is like any other disease (ii) Psychosocial support	HIV/AIDS perceived as not a serious threat	Acceptance of the illness	
*…I didn't tell. And my close relatives, I fear they would tell others and let the information spread and it could result in discrimination.*	(i) Comfort (ii) Relatives tell others about HIV status	Social support groups serve as forum for learning and exchange of information	Role of social capital	
